# Chronic Stress and the Brain–Kidney Axis in Renal Injury: From Neuroendocrine Dysregulation to Kidney Damage

**DOI:** 10.3390/ijms27188356

**Published:** 2026-09-19

**Authors:** Chunyun Zhang, Cheng Wan, Jing Sun, Zhiwen Wang, Qian Yuan, Chun Zhang

**Affiliations:** Department of Nephrology, Union Hospital, Tongji Medical College, Huazhong University of Science and Technology, Wuhan 430022, China; chunyunzhang@hust.edu.cn (C.Z.); stellarwane@126.com (C.W.); sunjingde1101@126.com (J.S.);

**Keywords:** chronic stress, brain–kidney axis, mineralocorticoid receptor, glucocorticoids, chronic kidney disease

## Abstract

Chronic stress is a pervasive psychophysiological burden and an independent risk factor for chronic kidney disease (CKD). Large-scale cohort studies confirm that stress exposure significantly increases CKD risk and accelerates renal decline, even after adjusting for traditional confounders. However, the neuroendocrine mechanisms translating stress into direct kidney damage remain unclear. This review focuses on the brain–kidney axis, a bidirectional framework integrating neural, endocrine, and immune signals. Under chronic stress, sustained overactivation of the hypothalamic–pituitary–adrenal (HPA) axis and sympathetic nervous system (SNS), coupled with secondary renin–angiotensin–aldosterone system (RAAS) dysregulation, creates a deleterious intrarenal milieu. Unlike classical models driven by high salt or metabolic disorders, a proposed hallmark of stress-induced injury is the non-classical activation of the mineralocorticoid receptor (MR). Given MR’s equal affinity for cortisol and aldosterone, the protective barrier maintained by 11β-hydroxysteroid dehydrogenase type 2 (11β-HSD2) is compromised under persistent glucocorticoid elevation. This may allow cortisol to escape inactivation and aberrantly activate intrarenal MR, a process putatively termed gluco-mineralo cross-activation. This mechanism is hypothesized to initiate profibrotic cascades via NADPH oxidase 4 (NOX4)/ROS/TGF-β/Smad pathways and amplifies the complement C5a-C5a receptor 1 inflammatory axis, culminating in fibrosis and sclerosis. Based on these insights, we propose stress-related kidney disease (SRKD) as a distinct clinical entity. This review synthesizes current evidence to guide precision interventions targeting the brain–kidney axis.

## 1. Introduction

Chronic stress has emerged as a pervasive psychophysiological burden in modern society. Beyond its established role in cardiovascular and metabolic disorders, a growing body of epidemiological evidence identifies chronic stress as an independent contributor to the onset and progression of chronic kidney disease (CKD). Landmark cohort studies, including the Swedish Stress Related Disorders Study (SCREAM) [1] and the UK Biobank [2], have demonstrated that exposure to severe stress or adverse life events significantly increases the risk of incident CKD and accelerates renal function decline, even after rigorous adjustment for traditional confounders such as hypertension, diabetes, and lifestyle factors.

Despite these robust clinical associations, the specific pathophysiological mechanisms linking the brain to kidney damage remain incompletely understood. The brain–kidney axis provides a crucial framework for understanding this connection. This bidirectional regulatory circuit integrates central neural pathways, neuroendocrine outputs, and renal afferent signaling to maintain homeostasis. Under chronic stress, this axis becomes dysregulated. Sustained activation of the hypothalamic–pituitary–adrenal (HPA) axis and the sympathetic nervous system (SNS), coupled with secondary activation of the renin–angiotensin–aldosterone system (RAAS), creates a deleterious neuroendocrine milieu within the kidney.

A key distinction between stress-related kidney injury and classical CKD subtypes lies in the modulation of the mineralocorticoid receptor (MR). In the kidney, MR possesses an equal affinity for aldosterone and cortisol. Under physiological conditions, 11β-hydroxysteroid dehydrogenase type 2 (11β-HSD2) acts as a protective barrier, inactivating cortisol to ensure MR selectivity for aldosterone. However, chronic stress disrupts this barrier. Persistently elevated glucocorticoids can saturate or downregulate 11β-HSD2, allowing cortisol to gain access to MR—a phenomenon provisionally termed gluco-mineralo cross-activation. This non-classical MR activation is hypothesized as the molecular hallmark of stress-induced renal damage, although direct in-human validation is still lacking.

**Figure 1:**
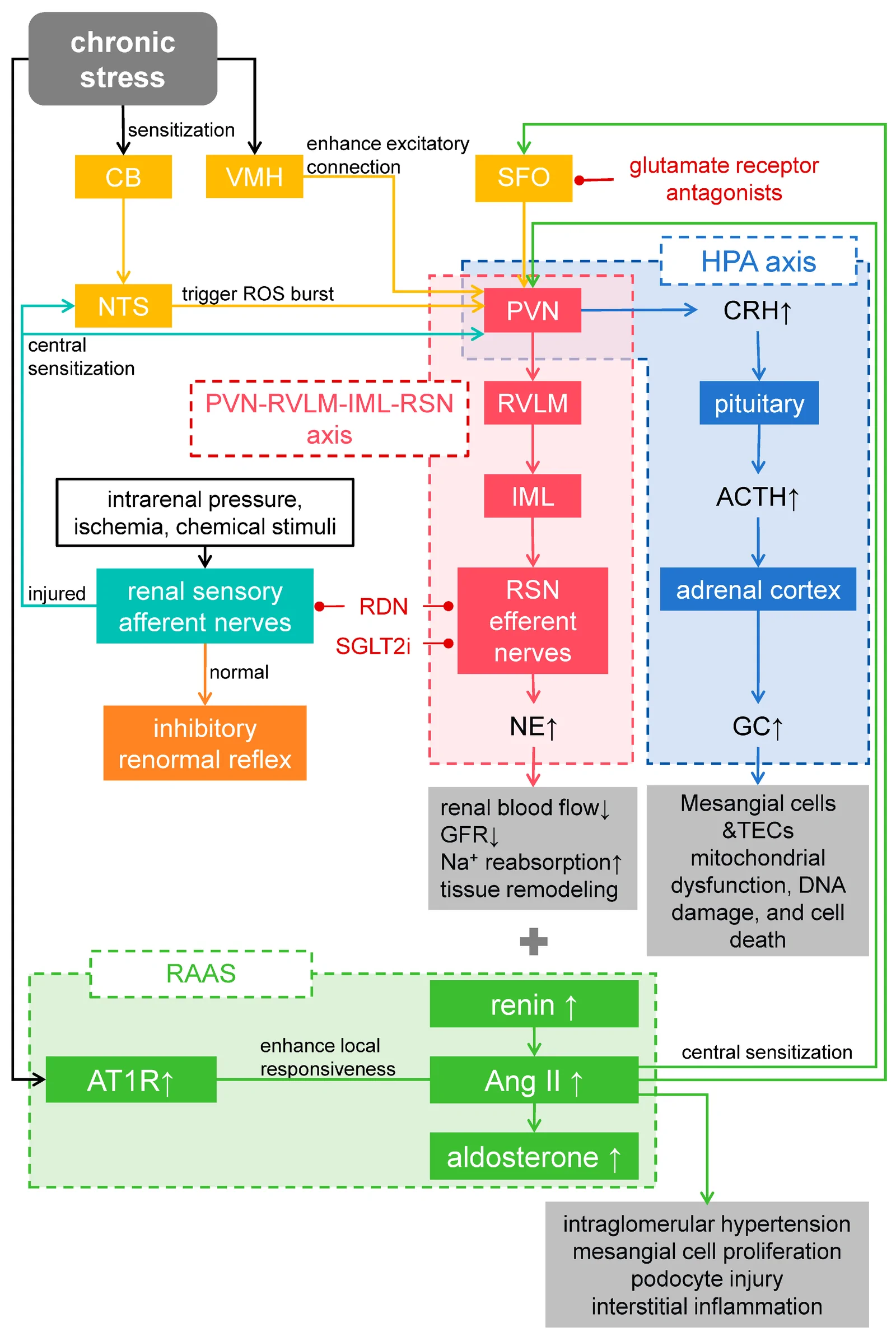
**Schematic of the brain–kidney axis and chronic stress-induced renal injury pathways.** The efferent pathway (VMH-PVN-RVLM-IML-RSN) stimulates NE release which reduces renal blood flow and GFR, and contributes to reabsorption of sodium ion, leading to renal tissue remodeling. Chronic stress enhances this drive via increased VMH-PVN input and SFO-mediated humoral-to-neural conversion (e.g., Ang II). The afferent pathway conveys renal sensory signals to central nuclei, forming a renorenal reflex loop that serves a homeostatic inhibitory function under physiological conditions, but switches to an excitatory drive upon renal injury, promoting a vicious cycle of sympathetic overactivation and progressive damage. At the molecular level, chronic stress activates the HPA axis which promotes CRH, ACTH and GC release. And chronic stress also activates RAAS which promotes activation of renin and Ang II, and finally upregulates aldosterone. Upregulated aldosterone contributes intraglomerular hypertension, mesangial cell proliferation, podocyte injury and interstitial fibrosis. At the same time, increased aldosterone also stimulates the efferent pathway (VMH-PVN-RVLM-IML-RSN). Upregulated GC and activated RAAS synergistically promote renal injury. Interventions targeting distinct nodes of this axis—including glutamate receptor antagonists (blocking SFO-PVN glutamatergic transmission), SGLT2 inhibitors (modulating renal sympathetic reflex sensitivity), and RDN (blocking renal sympathetic nerve activity)—have been shown to suppress this maladaptive cycle. Abbreviations: ACTH, adrenocorticotropic hormone; Ang II, angiotensin II; AT1R, angiotensin II type 1 receptor; CB, carotid body; CRH, corticotropin-releasing hormone; GC, glucocorticoid; GFR, glomerular filtration rate; HPA, hypothalamic–pituitary–adrenal axis; IML, intermediolateral column; NE, norepinephrine; NTS, nucleus tractus solitarius; PVN, paraventricular nucleus; RAAS, renin–angiotensin–aldosterone system; RDN, renal denervation; ROS, reactive oxygen species; RSN, renal sympathetic nerve; RVLM, rostral ventrolateral medulla; SFO, subfornical organ; SGLT2i, sodium–glucose cotransporter 2 inhibitor; TEC, tubular epithelial cell; VMH, ventromedial hypothalamus.

This non-classical activation triggers potent downstream effectors, including the NADPH oxidase 4 (NOX4)/reactive oxygen species (ROS)/transforming growth factor-beta (TGF-β)/Sma- and Mad-related protein (Smad) fibrotic cascade and the complement C5a-C5a receptor 1 (C5aR1) inflammatory axis, ultimately driving tubulointerstitial fibrosis and glomerular sclerosis. Based on these mechanistic insights and epidemiological findings, we propose the working concept of stress-related kidney disease (SRKD) as a distinct clinical entity. This review synthesizes current evidence linking chronic stress to renal injury through the brain–kidney axis, details the putative molecular pathways of glucocorticoid-mediated MR activation, and explores emerging therapeutic strategies, aiming to provide a theoretical basis for the precision management of SRKD (Figure 1).

## 2. Epidemiological Evidence Linking Chronic Stress and Kidney Disease

### 2.1. Definition of Chronic Stress and Epidemiological Evidence

Chronic stress is a pathophysiological state in which an individual’s adaptive responses fail under long-term or repeated psychological, social, or environmental stressors. This failure leads to persistent activation of the HPA axis and the SNS. This sustained neuroendocrine activation can cause abnormal cortisol secretion rhythms, increased sympathetic tone, and immune–inflammatory imbalance. Several large population-based cohort studies provide evidence for the association between psychological stress and kidney injury risk. The SCREAM cohort included 30,998 patients with stress-related disorders (SRDs) and 116,677 matched controls [1]. Over a median follow-up of 3.2 years, SRD patients had a 23% higher risk of CKD progression (hazard ratio [HR] 1.23, 95% confidence interval [CI] 1.10–1.37) and a 22% higher risk of acute kidney injury (HR 1.22, 95% CI 1.04–1.42) [1]. The UK Biobank study (N = 140,997, median follow-up 5.98 years) further showed that continuous exposure to adverse life events from childhood to adulthood increased CKD risk by 37% (HR 1.37, 95% CI 1.14–1.65) [2]. Body mass index (BMI), smoking, and hypertension partially mediated this association, which was enriched in the interleukin-1 receptor/inflammatory pathway [2]. In addition, a study based on the Heart and Soul cohort (including Veterans Affairs participants) showed that patients with post-traumatic stress disorder (PTSD) had a faster annual estimated glomerular filtration rate (eGFR) decline (PTSD group 2.97 vs. control group 2.11 mL/min/1.73 m^2^/year). PTSD was independently associated with rapid eGFR decline (>3 mL/min/1.73 m^2^/year) (odds ratio [OR] 1.91, 95% CI 1.12–2.25). This association remained consistent after adjusting for hypertension, diabetes, depression, and psychiatric medications (OR 1.98, 95% CI 1.00–3.77) [3].

It should be noted that the operational definitions of chronic stress vary across these studies. The SCREAM cohort used clinically diagnosed SRD as the exposure. The UK Biobank used cumulative scores of adverse life events. The Heart and Soul cohort focused on PTSD. These three operational definitions reflect a continuum from clinical disease to psychosocial exposure. Their heterogeneity is both a challenge to evidence strength and an indication that stress-related kidney injury may exist across multiple dimensions, from subclinical to clinical. Together, these studies suggest that chronic psychological stress is not only an independent risk factor for CKD but also that its cumulative effects significantly increase disease burden.

### 2.2. Proposal and Controversies Surrounding the Concept of Stress-Related Kidney Disease

Based on the epidemiological clues above, there is sufficient evidence to consider chronic stress as an independent pathogenic factor in CKD. However, no corresponding nephrological entity has been formally named. This review proposes stress-related kidney disease (SRKD) as a working concept. It describes a kidney injury phenotype driven primarily by chronic psychological or social stress, with or without the superimposition of traditional risk factors such as hypertension, diabetes, and obesity. Its operational definition could be provisionally stated as a subset of kidney injury in which chronic stress exposure (SRD diagnosis, cumulative adverse life events, PTSD, etc.) is independently associated with CKD incidence or progression after excluding or adjusting for traditional risk factors. It is important to emphasize that SRKD has not yet been included in the International Classification of Diseases (ICD) or Kidney Disease: Improving Global Outcomes classification systems. Its clinical boundaries, diagnostic criteria, and distinction from non-stress CKD remain controversial. The core of the controversy is whether chronic stress directly causes structural kidney damage or acts indirectly by mainly exacerbating intermediary factors such as hypertension, diabetes, and inflammation. The cohorts of Su et al. [1] and Li et al. [2] still showed significant HRs after adjusting for hypertension, diabetes, BMI, and smoking, suggesting pathways independent of traditional risk factors. However, the mediation proportions reported by Li et al. (BMI, smoking, and hypertension as partial mediators) also indicate that indirect pathways cannot be ignored [2]. Proponents of a direct pathogenic mechanism argue that chronic stress can drive glomerulosclerosis and tubulointerstitial fibrosis through neuroendocrine pathways, including sustained HPA activation leading to cortisol abnormalities, sympathetic overactivation causing renal vasoconstriction and the RAAS activation, and non-classical MR activation (gluco-mineralo cross-activation), rather than merely amplifying traditional risk factors [4,5,6]. Critics, however, point out that most current evidence comes from observational cohorts and animal models, making it difficult to completely exclude residual confounding. The causal chain between stress and kidney injury still requires prospective interventional studies, for example, randomized controlled trials (RCTs) targeting HPA, RAAS, and sympathetic pathways, for confirmation. The brain–kidney axis, as a bidirectional regulatory framework integrating neural, endocrine, and immune signals, provides a key theoretical tool for addressing this controversy.

In addition to direct neuroendocrine pathways, chronic stress may also indirectly exacerbate kidney damage through unhealthy lifestyle behaviors. An epidemiological study by Li et al. found that BMI, smoking, and hypertension partially mediate the association between stress and CKD [2], indicating that behavioral factors play a significant role in residual risk. Among these, three key mediators are particularly relevant: (i) high-salt diet, promoted by stress-induced increased appetite and dysregulation of reward circuits, enhances activation of the RAAS and may further impair intrarenal 11β-HSD2, lowering the cortisol escape threshold and promoting cross-activation of glucose and salt metabolism; (ii) physical inactivity, driven by stress-related fatigue and anhedonia, diminishes the protective effects of regular exercise on the kidneys and accelerates CKD progression via insulin resistance and chronic inflammation; (iii) smoking, closely linked to stress exposure, induces oxidative stress and endothelial dysfunction, potentially synergizing with the NOX4/ROS fibrosis pathway. These indirect pathways do not act in isolation but form feedback loops with direct neuroendocrine mechanisms—for example, high-salt intake and sedentary behavior promote obesity and hypertension, which in turn further activate RAAS and sympathetic outflow. Recognizing these behavioral mediators offers opportunities for lifestyle interventions that could partially mitigate stress-related kidney injury, while also paving the way for future pharmacotherapies targeting the brain–kidney axis.

The following sections will start from its neuroanatomical basis and systematically describe how chronic stress translates into kidney injury through this axis.

To enhance the clinical application value of this working concept, we have proposed a preliminary operational framework for SRKD based on existing evidence: (1) Quantification of chronic stress: It should be combined with psychological measurement tools and neuroendocrine studies. Psychological screening can use the Perceived Stress Scale-10 (PSS-10), or follow the diagnosed stress-related disorders (such as the types defined in the SCREAM cohort). Neuroendocrine confirmation requires the use of objective biomarkers, such as the flattening of the circadian rhythm of salivary cortisol (i.e., the cortisol level before sleep is more than 50% higher than during wakefulness), or an increase in the ratio of low frequency/high frequency in heart rate variability, indicating dysfunction of the HPA axis and the SNS. (2) Attribution of renal injury to stress: For patients with traditional risk factors, if the degree of renal injury is not proportional to the severity or duration of these factors, or if eGFR rapidly decreases and albuminuria significantly increases after a major stress event, it can be considered SRKD. Adjusting for factors such as hypertension, diabetes, and BMI (as in the methods used in the SCREAM and UK Biobank studies) helps to identify the independent contribution of stress. (3) Exclusion of other kidney diseases: SRKD should only be considered after reasonable exclusion of common primary kidney diseases (such as IgA nephropathy (IgAN), diabetic nephropathy, and hypertensive nephrosclerosis) through clinical, laboratory, or histological examinations. Therefore, SRKD is an exclusionary diagnosis used to describe the damage caused by stress, which cannot be fully explained by known CKD subtypes. This framework is intended as a starting point for clinical research and requires prospective validation.

## 3. Neuroanatomical and Physiological Basis of the Brain–Kidney Axis

### 3.1. Efferent Pathways: Central Neural Control of Renal Sympathetic Nerves

The hypothalamic paraventricular nucleus (PVN) serves as a higher-order center for autonomic regulation and plays a central role in integrating stress responses and modulating sympathetic output. Corticotropin-releasing hormone (CRH) neurons and glutamatergic neurons in the PVN project downward to form synaptic connections with premotor sympathetic neurons in the rostral ventrolateral medulla (RVLM). RVLM axons then descend to the intermediolateral column (IML) of the spinal cord at the thoracolumbar level, where they synapse with preganglionic sympathetic neurons. Preganglionic fibers eventually innervate the kidney via the splanchnic nerves and the renal plexus, forming the PVN-RVLM-IML-renal sympathetic nerve (RSN) efferent axis [7,8]. The anatomical and functional basis of this axis has been systematically elucidated by DiBona [7] and Xiong et al. [8] in models of hypertension and renal sympathetic remodeling.

It is worth noting that chronic intermittent hypoxia (CIH), a core pathology of obstructive sleep apnea, acts as a form of physiological chronic stress and can activate RSN via upstream sensory receptors along this axis. In an 8-week CIH rat model, Liu et al. [9] demonstrated that CIH induced sensory plasticity in the carotid body (CB) (upregulation of tyrosine hydroxylase [TH] and vesicular monoamine transporter 2 [VMAT2]). Following CB sensitization, signals were projected through the nucleus tractus solitarius (NTS) to the PVN. This triggered a serotonin (5-HT)/5-HT2A receptor (5-HT2AR)/protein kinase C-signaling-dependent NOX4-mediated ROS burst in the PVN, which in turn enhanced RSN firing and elevated blood pressure. Intracranial injection of Tempol (an antioxidant) or CB resection partially reversed these changes [9]. The value of this study lies in embedding the upstream link of CB sensitization to PVN (ROS) to RSN into the classical PVN-RVLM-IML-RSN axis. This suggests that chronic stress-like stimuli (hypoxia, sleep fragmentation, psychological stress) may share a common central-sympathetic output node, namely, redox and 5-HT2AR signaling in the PVN. This finding mechanistically echoes the central theme of this review, namely, psychological or physiological chronic stress leading to brain–kidney axis dysregulation and subsequently kidney injury.

When RSN terminals release norepinephrine, it acts on β1-adrenergic receptors on effector cells within the kidney, including renal vascular smooth muscle cells, juxtaglomerular granular cells, and renal tubular epithelial cells. This regulates renal blood flow, GFR, sodium reabsorption, and renin release [7]. During acute stress, RSN discharge can increase several-fold, causing intense renal vasoconstriction and a transient drop in GFR. Chronic sustained sympathetic activation induces renal tissue remodeling (increased perivascular nerve fiber density around renal arteries and enlargement of nerve ganglia), driving long-term structural and functional damage. The following section will further discuss the afferent limb of the brain–kidney axis, forming the renorenal reflex loop via renal sensory input back to the central nervous system.

### 3.2. Afferent Pathways and Detailed Circuitry Analysis

The kidney is not merely a passive recipient of central neural regulation. It also sends mechanical and chemical information from the local renal environment back to the central nervous system via abundant renal sensory afferent nerves, forming a renorenal reflex closed loop. Afferent fibers are primarily distributed in the renal pelvic wall and around renal blood vessels. They can sense changes in intrarenal pressure, ischemia, and chemical stimuli such as prostaglandins, adenosine, and bradykinin. Upon stretch activation, they reduce contralateral renal sympathetic output via an inhibitory renorenal reflex, coordinating sodium and water excretion between the two kidneys [10]. When nephrons are damaged or intrarenal pressure rises, these afferent signals travel via spinal cord pathways and ultimately project to key autonomic control centers such as the NTS and the PVN [7]. This afferent pathway endows the kidney with a dual identity as a sensory organ. When kidney injury occurs, afferent signals can trigger enhanced central sympathetic outflow, leading to increased RSN efferent activity. This further exacerbates renal vasoconstriction, promotes renin release, and causes sodium/water retention, forming a vicious cycle of kidney injury to sympathetic activation to worsening kidney injury [7]. Classic foundational work by DiBona et al. demonstrated that selective ablation of renal afferent nerves, via surgical or pharmacological methods, could effectively break this vicious cycle. This significantly reduced renal fibrosis and blood pressure elevation in animal models of CKD [7]. Furthermore, clinical studies have observed enhanced substance P immunoreactivity in renal nerves of hypertensive patients, suggesting that changes in sensory nerve components and enhanced afferent signals play an important role in the progression of hypertension and kidney injury [8].

In recent years, with the application of advanced techniques such as neural tracing and optogenetics, our understanding of the brain–kidney regulatory circuitry has reached the level of fine synaptic connections. Recent studies have found that the subfornical organ (SFO), a circumventricular sensory structure, can sense circulating humoral signals such as osmotic pressure and angiotensin II (Ang II). It activates parvocellular neurons in the PVN via glutamatergic projections. This humoral–neural conversion mechanism has been confirmed by experiments showing that SFO stimulation by Ang II drives renal sympathetic activity through PVN glutamate receptors [11,12]. The resulting SFO-PVN connection is a critical node for converting humoral signals into neural activity. Activated PVN neurons then drive RVLM sympathetic neurons via descending projections, ultimately affecting kidney function through RSN, forming the SFO-PVN-RVLM multisynaptic pathway. In 2023, Cao et al. [12], using transsynaptic viral tracing combined with optogenetics, demonstrated for the first time that this multisynaptic circuit is hyperactivated in models of chronic kidney injury and heart failure. This hyperactivation caused abnormally elevated RSN firing frequencies, which could not be fully explained by blood pressure changes. This finding suggests it may be a direct neurogenic driver of kidney injury [12]. Interventions targeting this circuit, such as local injection of glutamate receptor antagonists in the SFO to block excitatory input, or RDN to cut RSN efferent/afferent fibers, suppressed the hyperactivation and reduced structural damage to both the kidney and heart [12]. Notably, RDN has been independently shown to reduce T-cell infiltration, dendritic cell activation, and levels of interleukin-6 and isoketal adducts (oxidative stress markers) in renal tissue [13]. This is mutually corroborative with the circuit hyperactivation to sympathetic drive to kidney injury mechanism revealed by Cao et al. [12]. These findings not only establish the key role of the SFO-PVN-RVLM circuit in the brain–kidney axis but also provide a theoretical foundation for developing novel therapeutic strategies targeting this circuit.

It should be noted that many of the studies cited in this section employed models of known renal injury or heart failure, where the main stress factors were hemodynamic and metabolic abnormalities rather than purely psychological stress. Although these models are helpful for in-depth understanding of the non-adaptive renal remodeling response and the mechanism of central sympathetic nerve amplification, caution is still needed when applying the results to kidney diseases related to stress. Future studies using specific psychological stress patterns (such as chronic mild stress, social frustration) are needed to verify the direct effect of psychosocial stress factors on the dysfunction of the brain–renal axis.

## 4. Molecular Mechanisms of Chronic Stress Modulation of the Brain–Kidney Axis

### 4.1. Sustained HPA Axis Activation and Cortisol-Mediated Injury

Under chronic stress, the PVN releases increased CRH, which stimulates the anterior pituitary to secrete adrenocorticotropic hormone. This leads to sustained glucocorticoid secretion from the adrenal cortex (cortisol in humans; corticosterone in rats), disrupting normal circadian rhythms and negative feedback regulation. Studies show that rats exposed to chronic mild stress (CMS) have significantly elevated serum corticosterone levels with disrupted circadian rhythms. This hypercorticosterone state persists throughout the stress period. After stress cessation, circadian rhythms can recover, suggesting that HPA axis activation during CMS is state-dependent rather than trait-dependent [14]. As a potent glucocorticoid, cortisol acts directly on glomerular mesangial cells and renal tubular epithelial cells. It induces mitochondrial bioenergetic dysfunction, DNA damage, and cell death via glucocorticoid receptor (GR) activation [15]. Notably, under chronic stress, GR dysregulation is closely linked to increased FK506-binding protein 5 (FKBP5) protein expression. FKBP5 inhibits GR nuclear translocation, thereby weakening negative feedback and sustaining HPA axis hyperactivity [16]. This process exhibits sex differences, with female rats showing higher HPA axis sensitivity to chronic stress. FKBP5 is involved in this sex difference, possibly through estrogen-mediated modulation of GR signaling [17]. These findings reveal the molecular mechanism by which chronic stress, through sustained HPA axis activation, leads to cortisol-mediated kidney injury. The cortisol output from the HPA axis, together with aldosterone from the RAAS (discussed in the next section), constitutes the ligand sources for MR, laying the foundation for subsequent non-classical MR activation.

It should be noted that the interaction between FKBP5 and the GR signaling pathway is regulated by estrogen, which may lead to sex-specific hypothalamic–pituitary–adrenal axis responses under chronic stress. Compared to male rats, female rats exhibit higher hypothalamic–pituitary–adrenal axis sensitivity and changes in FKBP5 expression, which may be due to the enhanced GR signaling pathway mediated by estrogen. However, most preclinical studies on stress-related kidney diseases are conducted in male animals, and the effects of estrogen or gender differences on the cross-activation of glucocorticoids and mineralocorticoids within the kidneys have largely not been deeply explored. Future research should include both sexes and investigate whether the estrogen status (such as ovariectomy, estrogen replacement) will affect the development of secondary glomerulonephritis (SRKD), in order to promote targeted gender-specific intervention measures.

### 4.2. Secondary RAAS Activation and Excessive Renin–Angiotensin Drive

Chronic stress directly stimulates renin release from juxtaglomerular cells via sympathetic activation, initiating the RAAS cascade. Renin catalyzes the conversion of angiotensinogen to angiotensin I (Ang I), which is then converted to Ang II by angiotensin-converting enzyme. Ang II subsequently stimulates aldosterone secretion from the adrenal cortex. Studies show that CMS male rats exhibit significantly elevated plasma RAAS activity. Heart rate variability (HRV) analysis reveals that long-term stress leads to simultaneous enhancement of sympathetic and parasympathetic activity without disrupting autonomic balance. Cardiac angiotensin type 1 receptor (AT1R) messenger RNA (mRNA) was upregulated, suggesting that CMS drives cardiac and circulating RAAS activation via sympathetic–RAAS coupling [18]. Concurrent studies further showed that CMS upregulates AT1a receptor mRNA expression in the renal medulla, enhancing local renal tissue responsiveness to Ang II [19]. Ang II, as a potent vasoconstrictor and pro-inflammatory factor, induces intraglomerular hypertension, mesangial cell proliferation, and podocyte injury. It also activates the renal tubular nuclear factor-kappa B (NF-κB) pathway, triggering interstitial inflammation. These non-hemodynamic effects are core mechanisms of RAAS-mediated kidney injury [20]. In the context of diabetic kidney disease, a hyperglycemic environment upregulates renal MR protein and mRNA expression, making renal tissue hyper-responsive to even normal circulating aldosterone concentrations. This phenomenon is termed aldosterone sensitivity enhancement [21]. CMS-induced RAAS activation is closely linked to cardiac autonomic dysfunction, with the two mutually amplifying each other and further exacerbating kidney injury [18]. These findings emphasize the critical role of secondary RAAS activation in chronic stress-induced kidney injury. In patients with comorbid diabetes, the above RAAS activation synergizes with upregulated renal MR, allowing cortisol from the HPA axis and aldosterone from the RAAS to converge on intrarenal MR. This sets the stage for non-classical MR activation, detailed in Section 4.

### 4.3. Sympathetic Overactivation: Bidirectional Central–Renal Amplification

Under chronic stress, the ventromedial hypothalamus (VMH), a key brain region integrating stress information and energy homeostasis, receives enhanced excitatory input. This strengthens VMH to PVN connectivity, driving PVN sympathetic output and increasing RSN firing frequency. This pathway is particularly prominent in chronic stress-induced sympathetic overactivation [22]. Simultaneously, CMS in male rats for 4 weeks upregulates AT1a receptor mRNA expression in both the hypothalamus/thalamus diencephalon and the renal medulla, suggesting coordinated upregulation of central and local renal RAAS under chronic stress [19]. CMS male rats also show elevated plasma RAAS activity, upregulated cardiac AT1R mRNA, increased heart weight, and enlarged cardiomyocyte cross-sectional area. HRV analysis showed elevated low frequency (LF), high frequency (HF), and LF/HF ratio, with both sympathetic and parasympathetic activity enhanced without disrupting autonomic balance [18]. Notably, in a rabbit model of diabetes, the SGLT2 inhibitor empagliflozin, without lowering blood pressure, restored the abnormally enhanced renal sympathetic baroreflex sensitivity (maximal renal sympathetic nerve activity [RSNA] response to hypotension) to non-diabetic levels. This suggests it modulates the renal sympathetic reflex arc through natriuresis-related mechanisms [23]. CMS-induced sympathetic overactivation and increased RAAS activity act synergistically to drive cardiac structural and functional changes [18].

The above pathways do not operate independently in parallel. Ang II from the RAAS can enhance PVN sympathetic output (central sensitization), while sympathetic activation stimulates renin release (peripheral amplification), forming a positive feedback loop. The MR, a core node in this loop, will be detailed in the next section.

## 5. Molecular Pathways: Non-Classical MR Activation and Downstream Cascades

### 5.1. MR Distribution in the Kidney and Ligand Selectivity

MR distribution in the kidney is highly cell-type specific. Its expression is widespread in renal tubular epithelial cells (most abundant in aldosterone-sensitive distal nephron/collecting duct principal cells), glomerular mesangial cells, podocytes, and infiltrating non-epithelial cells such as macrophages [24,25,26]. This broad distribution determines MR’s multiple roles in renal physiology and pathology. Under normal conditions, the main natural ligand for MR is aldosterone. However, MR has similar affinity for cortisol (human; corticosterone in rats). In aldosterone-sensitive distal nephron segments, 11β-HSD2 serves as a critical gatekeeper. It oxidizes cortisol to inactive cortisone (11-dehydrocorticosterone in rats), preventing cortisol from binding to MR [27]. Thus, under physiological conditions, MR’s selective activation depends on aldosterone, and the mineralocorticoid activity of cortisol is shielded by the 11β-HSD2 barrier. Maintaining this ligand selectivity is fundamental to normal renal water–salt balance.

### 5.2. Gluco-Mineralo Cross-Activation: A Hallmark of Stress-Related Kidney Injury

Unlike kidney injury induced by metabolic syndrome or high-salt diets, the core feature of chronic stress-related kidney injury is the dual-axis (HPA and RAAS) synergistic drive of non-classical MR activation, where the weak mineralocorticoid activity of cortisol may be amplified when the 11β-HSD2 barrier is weakened [4,5]. In purely high-salt-induced models, MR activation mainly depends on aldosterone. In stress models, MR activation may involve dual contributions from both glucocorticoids and mineralocorticoids. This gluco-mineralo cross-activation is a potential molecular feature distinguishing stress-related nephropathy from other CKD subtypes [4,5].

Under chronic stress, persistently elevated circulating cortisol could theoretically saturate or downregulate 11β-HSD2 inactivation capacity, thereby gaining MR agonist activity and entering cells to activate MR. This glucocorticoid–mineralocorticoid cross-activation mechanism has been confirmed in adrenalectomized animals with exogenous glucocorticoid (GC) combined with 11β-HSD2 inhibition [4]. MR’s equally high affinity for cortisol/aldosterone and the conversion of cortisol to an MR agonist in the context of tissue injury have been confirmed in reviews [5]. However, it remains unclear whether intrarenal 11β-HSD2 activity is inhibited or whether direct cortisol–MR binding increases in CMS models, as direct enzyme activity and chromatin immunoprecipitation (ChIP) level evidence are lacking. Previous acute stress studies showed that renal 11β-HSD2 activity actually increases to protect MR selectivity, so the direction under CMS requires further verification. Animal studies suggest that MR target genes (e.g., serum- and glucocorticoid-regulated kinase 1, epithelial sodium channel subunit alpha) may be upregulated in stress-related kidney injury. Ras-related C3 botulinum toxin substrate 1, as a ligand-independent MR activator, can amplify MR signaling even under normal aldosterone conditions, exacerbating kidney injury [6,28].

**Figure 2:**
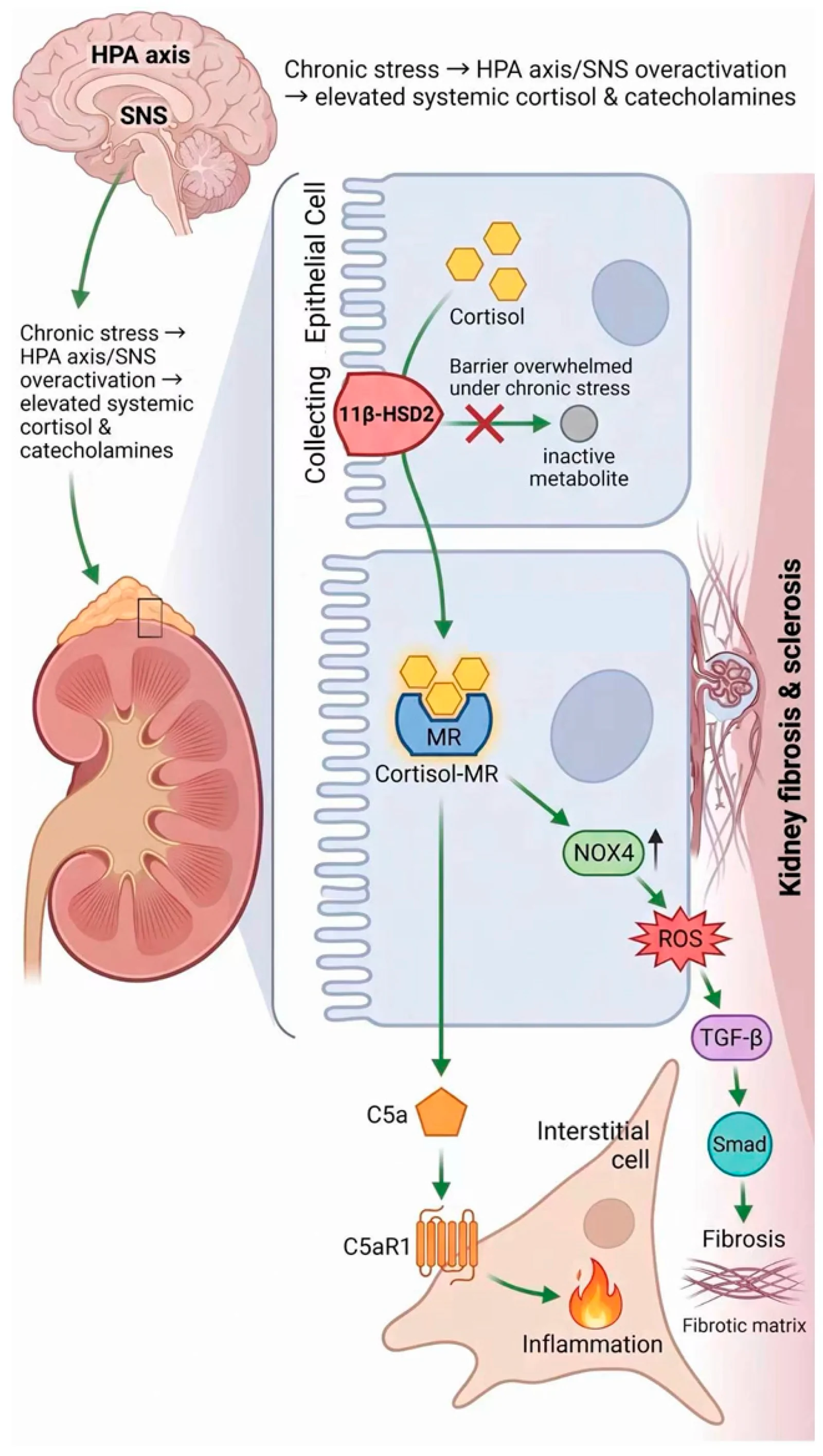
**The brain–kidney axis in stress-related kidney disease (SRKD).** Chronic stress overactivates the HPA axis/SNS, elevating systemic cortisol. In collecting epithelial cells, compromised 11β-HSD2 allows cortisol to aberrantly activate MR. Activated MR then upregulates the expression of NOX4 and the cascade reaction of ROS activation. This gluco-mineralo cross-activation drives NOX4/ROS/TGF-β/Smad-mediated fibrosis and C5a–C5aR1-driven in-flammation in interstitial cells, culminating in kidney fibrosis and sclerosis. Abbreviations: 11β-HSD2, 11β-hydroxysteroid dehydrogenase type 2; C5a-C5aR1, complement component 5a and complement component 5a receptor 1; MR, mineralocorticoid receptor; NOX4, NADPH oxidase 4; ROS, reactive oxygen species; Smad, Sma- and Mad-related protein; TGF-β, transforming growth factor-beta.

Future studies should directly verify changes in MR binding profiles in stress models through 11β-HSD2 activity assays and ChIP experiments. Such validation is critical before gluco-mineralo cross-activation can be firmly established as a clinical biomarker for SRKD. This potential gluco-mineralo cross-activation pattern provides a new molecular target for developing specific therapeutic strategies for stress-related nephropathy (Figure 2).

### 5.3. MR Activation Induces NOX4/ROS/TGF-β/Smad Fibrotic Pathways

MR activated by aldosterone or cortisol upregulates NOX4 expression through dual mechanisms, genomic effects (binding hormone response elements) and non-genomic effects (activating Src/epidermal growth factor receptor kinases). This leads to a sharp increase in intrarenal ROS production [29,30]. This ROS burst can activate latent TGF-β1 and upregulate TGF-β1 receptor expression, subsequently phosphorylating downstream Smad3 protein and initiating a pro-fibrotic transcriptional program. ROS derived from NOX4 plays a central role in TGF-β1-stimulated renal myofibroblast activation, acting downstream of Smad3 and upstream of extracellular signal-regulated kinase 1/2, forming the core of the NOX4/ROS/Smad3 cascade [31]. Aldosterone itself can upregulate intrarenal TGF-β1 expression via MR-dependent pathways [32]. TGF-β1 neutralizing antibodies completely block mesangial matrix expansion and renal dysfunction in db/db diabetic nephropathy models, confirming TGF-β1 as a key node in the renal fibrotic cascade [33]. At the mesangial cell level, aldosterone promotes fibronectin synthesis via the TGF-β1/Smad2 pathway and synergizes with TGF-β1 to inhibit matrix degradation and upregulate plasminogen activator inhibitor-1, amplifying pro-fibrotic effects [34,35]. Additionally, MR activation can induce osteopontin expression, exacerbating inflammatory responses and oxidative stress, thereby promoting renal interstitial fibrosis [36]. Together, these studies reveal the complete molecular mechanism by which MR activation drives renal fibrosis through the NOX4/ROS/TGF-β/Smad signaling cascade.

It is worth adding that MR activation may also contribute to CKD progression by amplifying the complement C5aR1 axis inflammatory signal. Recent research confirms that in diabetic nephropathy, the MR antagonist finerenone can inhibit the C5a-C5aR1 signaling pathway by downregulating guanine nucleotide-binding protein G(i) subunit alpha-2 (Gnai2/Gαi2), thereby reducing monocyte/tubular epithelial inflammation [37]. The pathogenic role of the C5a-C5aR1 axis in IgAN is supported by independent evidence. Respiratory syncytial virus (RSV) infection can amplify T helper 1/T helper 17 and suppress regulatory T cells through this axis, exacerbating IgAN kidney injury, effects reversed by C5aR1 blockade [38]. Intrarenal C5a/C5aR expression is positively correlated with IgAN pathological grade, proteinuria, and serum creatinine [39]. Notably, IgAN’s mucosa–bone marrow axis endows it with a unique infection–mucosal trigger phenotype [40]. Microbial triggers and mucosal immune tolerance imbalance are considered key initiating events [41]. These lines of evidence collectively suggest that MR antagonists, via the Gnai2-C5aR1 axis, may extend their therapeutic effects on complement-dependent inflammation from diabetic nephropathy to IgAN, which frequently involves infection–mucosal triggers. However, this cross-disease extrapolation requires direct validation in IgAN populations or models.

### 5.4. Future Research Directions of Glucocorticoid–Mineralocorticoid Cross-Activation

Although glucocorticoid–mineralocorticoid cross-activation has been proposed as a putative hallmark of SRKD, direct human evidence remains scarce, and several critical questions require further investigation. First, in-human validation of cortisol escaping 11β-HSD2 inactivation and binding to intrarenal mineralocorticoid receptors is currently lacking. Prospective studies should quantify urinary 11β-HSD2 activity and cortisol metabolites in SRKD patients, and ChIP assays on renal biopsies could confirm cortisol–MR binding while adjusting for traditional risk factors. Second, the directional regulation of 11β-HSD2 under chronic mild stress remains controversial. Longitudinal animal experiments measuring 11β-HSD2 mRNA, protein, and enzymatic activity, combined with human salivary cortisol rhythm assessments, are needed to clarify its regulatory mechanism. Third, high-salt intake, obesity, and diabetes impair 11β-HSD2 function and may confound stress-specific effects. Enrichment designs using neuroendocrine biomarkers (e.g., flattened salivary cortisol slope, elevated low-frequency/high-frequency ratio on heart rate variability) to pre-screen “high-stress” subgroups, together with mandatory adjustment for metabolic covariates, would help isolate the contribution of cross-activation. Fourth, no tool compound selectively blocks cortisol–MR activation without affecting aldosterone–MR signaling. Developing selective 11β-HSD2 modulators or cortisol-preferring MR antagonists via high-throughput screening is therefore a priority. Finally, SRKD lacks unified diagnostic criteria, and the prevalence of cross-activation in human CKD is unknown. Large cohorts (e.g., SCREAM, UK Biobank) should incorporate salivary cortisol rhythm and heart rate variability as stratification biomarkers, and subgroup analyses within ongoing finerenone trials (e.g., FIND-CKD) may indirectly validate this concept. Addressing these gaps is essential to establish glucocorticoid–mineralocorticoid cross-activation as a validated therapeutic target in SRKD.

## 6. Clinical Translation and Intervention Strategies

### 6.1. Clinical Evidence for Mineralocorticoid Receptor Antagonists (MRAs)

Non-classical MR activation is a key link in chronic stress-driven kidney injury, making MR antagonists a highly promising intervention strategy. Finerenone, a third-generation, highly selective, non-steroidal MRA, first demonstrated renal benefit in the FIDELIO-DKD trial in patients with type 2 diabetes and CKD (composite renal endpoint HR 0.82, 95% CI 0.73–0.93) [42]. Regarding safety, FIDELIO-DKD reported a discontinuation rate due to hyperkalemia of approximately 2.3% in the finerenone group. In contrast, traditional steroidal MRAs such as spironolactone have relatively higher hyperkalemia and discontinuation risks in similar populations. Multiple indirect comparisons and network meta-analyses indicate that finerenone has a more favorable hyperkalemia risk profile than spironolactone [42,43].

Notably, evidence for MRAs in non-diabetic CKD (ndCKD) is rapidly accumulating. This is particularly important for extrapolating interventions to stress-related kidney disease. FIND-CKD (NCT05047263) is the first global phase 3 RCT evaluating finerenone in ndCKD. It enrolled 1584 non-diabetic CKD patients from 24 countries (chronic glomerulonephritis 57.0%, including IgAN 26.3% and focal segmental glomerulosclerosis 13.6%; hypertensive/ischemic nephropathy 29.0%). Baseline eGFR was 46.7 mL/min/1.73 m^2^, and median urine albumin-to-creatinine ratio (UACR) was 818.9 mg/g. The primary endpoint was the eGFR slope over 32 months. Results were published in 2026. The study showed that the annualized eGFR decline rate over 32 months was −3.3 mL/min/1.73 m^2^ in the finerenone group versus −4.0 in the placebo group, a between-group difference of 0.7 mL/min/1.73 m^2^/year (95% CI 0.3–1.1, *p* < 0.001), demonstrating significantly slower renal function decline. For key secondary endpoints, finerenone reduced the risk of the cardiorenal composite endpoint by 23% (HR 0.77, 95% CI 0.60–0.99, *p* = 0.04). At month 6, UACR reduction from baseline was significantly greater in the finerenone group (approximately 35%). The discontinuation rate due to hyperkalemia was only 1.5%, indicating acceptable safety [44]. Concurrently, real-world studies from multiple Chinese centers were reported in 2025: Xu’s team (Zhongshan Hospital Xiamen University, n = 37, IgAN 54.1% + membranous 24.3%) showed that finerenone treatment for 12 months reduced UACR by 60.90% (membranous subgroup 75.70%, IgAN subgroup 40.26%), with stable eGFR and no significant increase in serum potassium [45]. Liu Lijun’s team at Peking University First Hospital reported that adding finerenone to renin–angiotensin system inhibitor (RASi) background therapy in primary IgAN reduced UACR by 54.2% at 3 months without negatively affecting eGFR [46]. Liu Shuangxin’s team at Guangdong Provincial People’s Hospital reported a UACR reduction of approximately 42.29% at 3 months in the IgAN subgroup [47]. A multicenter observational study from the Fourth Affiliated Hospital of Zhejiang University School of Medicine and others showed a 45.1% reduction in proteinuria (protein-to-creatinine ratio [PCR]) at 6 months with combination therapy in IgAN [48]. Membranous nephropathy cohorts also reported similar UACR reductions of 40–45% [49]. Mechanistically, Chen Min’s team in Cells 2025 revealed that finerenone could downregulate Gαi2 by inhibiting MR, thereby attenuating the C5a-C5aR1 complement axis in diabetic nephropathy models. This provides a mechanistic clue for finerenone’s potential benefit in complement-dependent nephropathies, though direct evidence in IgAN requires further validation [37].

The above evidence is highly relevant to the theme of stress-related kidney disease because IgAN itself has a hallmark phenotype of mucosal infection triggers. Sixty to eighty percent of macroscopic hematuria episodes are preceded by upper respiratory or gastrointestinal infections. The underlying mucosa–bone marrow axis (palatine tonsils/gut Peyer’s patch plasma cells producing galactose-deficient IgA1 to bone marrow homing to circulating immune complexes) is one of the four pillars of IgAN pathogenesis [40]. The association between psychological stress and IgAN is currently based mainly on clinical observations (higher self-reported major life events before flares, positive correlation between Perceived Stress Scale-10 [PSS-10] scores and proteinuria). Large-scale cohort evidence is not as robust as the two major SRD-CKD studies. Preclinical and mucosal immunity studies suggest a possible brain–mucosa–bone marrow axis bridge, namely, psychological stress to sympathetic increase to mucosal plasma cell activation to IgA-related product upregulation [50]. Therefore, IgAN substantially overlaps with stress-related kidney disease at the mucosal–infection trigger level and is a reasonable analogy at the psychological stress trigger level. Future studies could perform stress biomarker (cortisol rhythm, HRV) analyses in IgAN subgroups within FIND-CKD and other ndCKD trials to validate the specific benefit of MR antagonists in stress-related kidney injury.

To apply this concept to clinical practice, future trials should adopt enrichment design and pre-screen patients by combining neuroendocrine stress biomarkers. These biomarkers include: diurnal rhythm of salivary cortisol (e.g., a flattened slope indicates dysfunction of the HPA axis), elevated low-frequency/high-frequency ratio in heart rate variability (reflecting excessive activation of the SNS), and psychological measurement scores such as the Perceived Stress Scale-10. These biomarkers can serve as inclusion and stratification criteria to identify the “high-stress” subgroup most likely to exhibit gluco-mineralo cross-activation. Potential confounding factors must be strictly controlled: traditional CKD risk factors (such as hypertension, diabetes, BMI) should be adjusted as covariates; stress-related behaviors (such as smoking, sleep disorders) need to be quantified; and the use of drugs that affect neuroendocrine pathways (such as antidepressants, beta-blockers) should be balanced between groups or controlled in sensitivity analyses. This biomarker-based stratification method is crucial for bridging the gap between mechanism research findings and clinical evidence.

### 6.2. Renal Denervation (RDN) and Its Renoprotective Potential

Renal sympathetic overactivation is a core mechanism of chronic stress-induced kidney injury. RDN, as a direct intervention targeting sympathetic nerve activity, therefore shows unique renoprotective potential. In diabetic nephropathy and CKD animal models, both radiofrequency and ultrasound RDN significantly reduce renal tissue norepinephrine content and alleviate tubulointerstitial fibrosis and glomerulosclerosis. This protective effect was partially independent of blood pressure reduction in some models [51]. Yao et al. [51], in transgenic (mRen-2)27 rats and streptozotocin-induced type 1 diabetic nephropathy models, found that chronic bilateral renal denervation effectively reduced renal expression of TGF-β1, type I collagen, and type IV collagen proteins. It alleviated mesangial expansion and basement membrane thickening. They also confirmed that RDN could block the feedback hyperactivation of renal sympathetic efferent signals to the central nervous system (involving PVN-RVLM circuit regulation), thereby delaying renal function decline [51]. Although RDN has received clinical approval for treating resistant hypertension, its safety and efficacy in non-hypertensive CKD patients, particularly those with stress-related kidney disease, still require validation in large-scale prospective RCTs.

### 6.3. SGLT2i: Sympathetic Inhibition and Metabolic–Neural Dual Targeting

SGLT2is were initially introduced as glucose-lowering agents. Subsequent studies, however, have revealed kidney and cardiovascular protective effects beyond glycemic control. Modulation of the SNS is considered one key mechanism. Empagliflozin, dapagliflozin, and other SGLT2is block renal glucose reabsorption. From a broad pharmacological perspective, this is accompanied by altered sodium transport in the renal tubules, reduced Na+/K+-ATPase-related energy consumption and oxygen demand, and possible effects on renal afferent sympathetic signaling [23]. In a rabbit model of diabetes, empagliflozin restored the abnormally enhanced renal sympathetic baroreflex sensitivity to normal, normalizing the compensatory RSNA response to hypotension, and this effect was independent of blood pressure lowering [23]. Herat et al. [52] further proposed in their review that SGLT2is affect the brain–kidney axis through metabolic–neural cross-targets: they reduce renal sympathetic activity and renal afferent drive on one hand, and indirectly suppress central sympathetic output through metabolic improvements and central interactions on the other. This dual mechanism gives SGLT2is a unique advantage in regulating stress-related sympathetic overactivation. In clinical practice, combination therapy with SGLT2i and MRAs may produce synergistic effects, simultaneously exerting anti-inflammatory, anti-fibrotic, and sympathoinhibitory actions. This represents an integrated therapeutic direction for stress-related kidney disease.

### 6.4. Future Directions

With rapid advances in biotechnology, single-cell omics and spatial transcriptomics offer unprecedented resolution for dissecting the complex mechanisms of chronic stress-induced kidney injury. Single-cell transcriptomics can reveal the heterogeneity of intrarenal MR activation at the cellular level, for example, identifying macrophage subsets that specifically respond to MR signaling in stress models, providing a molecular basis for precise targeting of specific cell types. Multi-omics integration strategies, covering epigenomics, metabolomics, and proteomics, can help systematically identify key biomarkers and potential drug targets for chronic stress-induced kidney injury. In addition, gene editing or neuromodulation techniques targeting specific central nodes of the brain–kidney axis are currently in active preclinical exploration.

At the clinical translation level, we recommend using enrichment designs in clinical trials. These should incorporate neuroendocrine activation biomarkers into inclusion and stratification criteria, for example, disrupted salivary cortisol circadian rhythm, abnormally elevated plasma renin activity/aldosterone ratio, and increased HRV LF/HF ratio. This approach can precisely identify patient subgroups most likely to benefit from brain–kidney axis-targeted interventions. Such biomarker-driven stratification strategies can help bridge the gap between basic mechanistic research and clinical translation, promoting the evolution of stress-related kidney disease from a proof-of-concept to truly individualized precision therapy. Notably, non-psychosocial stressors such as heavy metals also activate NF-κB/MAPK inflammatory cascades in the brain “32805460, 39357438”, suggesting that future SRKD research could explore whether similar cellular pathways operate in the human kidney [53,54].

## 7. Conclusions

Chronic stress, through the synergistic activation of the HPA axis and the RAAS, drives excessive excitation of the bidirectional brain–kidney circuit, ultimately leading to renal inflammation and fibrosis. Non-classical MR activation, particularly gluco-mineralo cross-activation, is proposed to constitute the key molecular hub of this pathological cascade; however, direct evidence in human kidneys remains elusive. Population-based cohort evidence and basic research collectively support the validity of SRKD as an independent clinical entity. However, the field currently lacks unified diagnostic criteria and a pathological classification system.

At the clinical intervention level, finerenone, as a new-generation non-steroidal MRA, has demonstrated cardiorenal benefits in diabetic kidney disease (DKD). Ongoing phase 3 trials such as FIND-CKD are extending its evidence base to ndCKD and IgAN. The latter has a hallmark phenotype of mucosal infection triggers, and psychological stress may also indirectly participate in its course via the brain–mucosa–bone marrow axis, showing substantial overlap with the SRKD theme. RDN, by blocking excessively activated renal sympathetic efferents, provides an alternative treatment option for drug-resistant patients. SGLT2 inhibitors indirectly suppress brain–kidney axis overexcitation through metabolic–neural cross-targets, forming complementary intervention pathways.

However, it must be acknowledged that several critical evidence gaps remain in this field: (1) direct evidence for gluco-mineralo cross-activation in human kidneys is still insufficient; (2) the specific benefits of MRAs, RDN, and SGLT2is in pure stress-related kidney injury (i.e., non-DKD or non-hypertensive kidney disease with comorbid stress) require further validation; (3) prospective studies of biomarkers based on brain–kidney axis mechanisms to guide clinical decisions are still lacking.

SRKD, as a working concept proposed in this review, will ultimately depend on the accumulation of three types of evidence for its formal establishment: (i) stratified cohort studies based on stress biomarkers (cortisol rhythm, HRV, catecholamine profiles); (ii) validation of specific therapeutic efficacy in SRKD subgroups through interventional trials targeting the HPA axis, RAAS, and sympathetic pathways; and (iii) operational definition and formal inclusion by future KDIGO and international consensus organizations.

Closing these evidence gaps is the key to moving SRKD from concept to clinical practice. This translational process ultimately relies on the synergistic advancement of basic mechanistic research, clinical evidence accumulation, and technological innovation. The goal is to truly transform SRKD into a diagnosable, preventable, and treatable clinical entity.

## Data Availability

No new data were created or analyzed in this study.

## Abbreviations

The following abbreviations are used in this manuscript:

Ang I	Angiotensin I
Ang II	Angiotensin II
AT1R	Angiotensin type 1 receptor
BMI	Body mass index
C5aR1	C5a-C5a receptor 1
CB	Carotid body
CI	Confidence interval
CIH	Chronic intermittent hypoxia
CKD	Chronic kidney disease
CMS	Chronic mild stress
CRH	Corticotropin-releasing hormone
DKD	Diabetic kidney disease
eGFR	Estimated glomerular filtration rate
FKBP5	FK506-binding protein 5
GC	Glucocorticoid
GFR	Glomerular filtration rate
Gnai2/Gαi2	Guanine nucleotide-binding protein G(i) subunit alpha-2
GR	Glucocorticoid receptor
HF	High frequency
HPA	Hypothalamic–pituitary–adrenal
HR	Hazard ratio
HRV	Heart rate variability
IgAN	Immunoglobulin A nephropathy
IML	Intermediolateral column
LF	Low frequency
mRNA	Messenger RNA
MR	Mineralocorticoid receptor
MRA	Mineralocorticoid receptor antagonist
ndCKD	Non-diabetic chronic kidney disease
NF-κB	Nuclear factor-kappa B
NOX4	NADPH oxidase 4
NTS	Nucleus tractus solitarius
OR	Odds ratio
PCR	Protein-to-creatinine ratio
PSS-10	Perceived Stress Scale-10
PTSD	Post-traumatic stress disorder
PVN	Paraventricular nucleus
RAAS	Renin–angiotensin–aldosterone system
RASi	Renin–angiotensin system inhibitor
RCT	Randomized controlled trial
RDN	Renal denervation
ROS	Reactive oxygen species
RSN	Renal sympathetic nerve
RSNA	Renal sympathetic nerve activity
RVLM	Rostral ventrolateral medulla
SCREAM	Swedish Stress Related Disorders Study
SFO	Subfornical organ
SGLT2i	Sodium–glucose cotransporter-2 inhibitor
Smad	Sma- and Mad-related protein
SRD	Stress-related disorder
SRKD	Stress-related kidney disease
TGF-β	Transforming growth factor-beta
TH	Tyrosine hydroxylase
UACR	Urine albumin-to-creatinine ratio
VMAT2	Vesicular monoamine transporter 2
VMH	Ventromedial hypothalamus
11β-HSD2	11β-hydroxysteroid dehydrogenase type 2
5-HT	Serotonin
5-HT2AR	5-HT2A receptor
